# Retraction Note: Effect of perioperative infusion of Dexmedetomidine combined with Sufentanil on quality of postoperative analgesia in patients undergoing laparoscopic nephrectomy: a CONSORT-prospective, randomized, controlled trial

**DOI:** 10.1186/s12871-019-0688-8

**Published:** 2019-02-13

**Authors:** Fuxi Song, Chunmiao Ye, Feng Qi, Ping Zhang, Xuexiang Wang, Yanfeng Lü, Alejandro Fernandez-Escobar, Chao Zheng, Liang Li

**Affiliations:** 1 Department of Anaesthesiology, the Second Hospital of Shandong University, No. 247 Beiyuan Street, Jinan, 250033 China; 2 Department of Breast Surgery, the Second Hospital of Shandong University, No. 247 Beiyuan Street, Jinan, 250033 China; 3 Department of Anaesthesiology, Qilu Hospital of Shandong University, No. 107 Wenhua West Road, Jinan, 250012 China; 4 The Institute for Translational Medicine, the Second Hospital of Shandong University, No. 247 Beiyuan Street, Jinan, 250033 China; 5 Department of Anoproctology, the Second Hospital of Shandong University, No. 247 Beiyuan Street, Jinan, 250033 China; 6 Translational Research Program, University of Toronto, 27 King’s College Circle, Toronto, ON M5S 1A1 Canada

**Retraction Note: BMC Anesthesiol (2018) 18:145**

**https://doi.org/10.1186/s12871-018-0608-3**

The authors have retracted this article [1] because they have recently found that the clinical results for the dosage of postoperative opioids in some patients were inconsistent with the results presented. In light of these findings, the authors no longer have confidence in the accuracy of the reported data and the conclusions of the article and wish to redesign the programme of postoperative analgesia and re-analyse their work. The authors would like to apologise for any inconvenience. All authors agree to this retraction.
